# Automated indexing in MEDLINE and the Medical Text Indexer (MTI), 2000–2025: a scoping review

**DOI:** 10.5195/jmla.2026.2406

**Published:** 2026-07-01

**Authors:** Dean Giustini, Alexandre Amar-Zifkin, Eileen Chen, Janice Y. Kung

**Affiliations:** 1 dean.giustini@ubc.ca, Reference Librarian/Health Sciences Librarian, UBC Biomedical Branch Library, Faculty of Medicine, University of British Columbia Library, Vancouver, British Columbia, Canada; 2 alexandre.amar-zifkin@umontreal.ca, Librarian, Bibliothèque de la Santé, Université de Montréal, Montreal, Quebec, Canada; 3 eileen.chen@ucsf.edu, Clinical Research Librarian/Education and Research Librarian, UCSF Library, University of California, San Francisco, San Francisco, California, USA; 4 janice.kung@ualberta.ca, Health Sciences Librarian, Geoffrey and Robyn Sperber Health Sciences Library, University of Alberta Library, Edmonton, Alberta, Canada

**Keywords:** Automated indexing, Algorithmic indexing, Medical Text Indexer (MTI), Medical Subject Headings (MeSH), PubMed/MEDLINE Searching, National Library of Medicine (NLM), Neural Network, Search Filters

## Abstract

**Objectives:**

To synthesize and map literature on automated indexing of the biomedical literature, with a focus on the Medical Text Indexer (MTI) at the National Library of Medicine (NLM). We review the drivers, benefits, and challenges of automated indexing, evolution of the MTI from 2000–2025, and impacts on information retrieval in MEDLINE.

**Methods:**

We conducted a scoping review following the JBI Manual for Evidence Synthesis and reported findings using PRISMA-ScR and PRISMA-S. We searched several bibliographic databases, key journals, conference proceedings, and grey literature sources, with no restrictions on language or study design. Eligible publications were published 2000–2025 and focused on MTI development. Screening, data charting, and thematic analysis were conducted by multiple reviewers.

**Results:**

We included 64 publications, with most originating from the United States (n=53, 83%) and five from Canada (8%). Study methods included evaluation or comparative studies (65%), qualitative descriptions (25%), and mixed methods (11%). MTI evolved from a rules-based recommendation tool in 2002 to the neural network–based MTIX in 2024. Despite numerous enhancements to the MTI, human curation remains necessary for approximately one-third of records to correct inaccuracies, capture missed concepts, and address errors arising from figurative language or algorithmic biases.

**Conclusions:**

This review synthesizes twenty-five years of MTI research (2000–2025). Despite reduced indexing times and a markedly improved algorithm, the MTIX has not yet achieved full equivalence to human indexing. Our findings suggest searchers should watch for algorithmic ambiguities in their MEDLINE searching and adapt accordingly. Health sciences librarians should work with stakeholders, including authors, to shape future algorithmic indexing methods, outputs, evaluation and research.

## INTRODUCTION

The National Library of Medicine (NLM) has conducted research into automated and semi-automated indexing since the 1980s [1, 2]. NLM’s past indexing achievements are outlined in *A History of the National Library of Medicine*, and include John Shaw Billings’ Index Medicus in 1879, print indexes of the 20th century, and MEDLARS in the 1960s – NLM’s first computerized system for MEDLINE indexing and information retrieval [3].

Automated indexing has been defined as “the assignment of index terms to documents by means of a computer,” with some human intervention, or none at all [4, 5]. The term covers a spectrum of computational approaches from semi-automated methods with human oversight to fully automated processes [4, 5]. These approaches typically rely on algorithms, natural language processing (NLP), and machine learning models to perform automated indexing [5] (see Appendix A—Glossary of terms and abbreviations).

In response to an increasing volume of publications, NLM began testing text mining and NLP approaches in the 1990s [6, 7] to assist indexers in assigning medical subject headings (MeSH) to MEDLINE records. In 2002, NLM’s Indexing Initiative introduced the Medical Text Indexer (MTI) to human indexers as a MeSH recommendation tool. MTI recommendations were integrated into the Data Creation and Maintenance System (DCMS) and used by human indexers to evaluate and select the most appropriate MeSH terms for each article [8]. MTI was an automated tool that supported, rather than replaced, the expertise and oversight of highly skilled indexers [4].

In 2021, NLM announced a major transition to the rules-based MTIA (Auto) algorithm to automate MEDLINE indexing, with human curation “applied as indicated” to ensure accuracy and completeness of records [9]. By 2024, NLM had implemented the MTIX (Medical Text Indexer–Next Generation), which uses convolutional neural network technology, for fully automated indexing with curation staff reviewing MEDLINE records from high-impact subject areas, and frequently searched topics [10].

Despite decades of MTI development and an extensive body of research examining its enhancements, no prior scoping or systematic review has synthesized the evidence on MTI-related research [4]. This scoping review represents the first systematic effort by health sciences librarians (HSLs) to identify, map, and synthesize this literature. We trace the evolution of the MTI by analyzing the drivers, benefits, and challenges of automated indexing, as well as its downstream effects on MEDLINE searching and its impact on indexing accuracy and completeness. By situating the MTI within the broader historical and technological development of automated indexing, this review offers a focused, original contribution to the medical library and information science literature.

### Research Questions (RQs)

Four (4) research questions guided our review:

- **RQ1: Drivers, benefits and challenges of automated indexing:** What are the key drivers, benefits and challenges of automated indexing in MEDLINE?
- **RQ2: Mapping evolution and performance:** What tools and technologies are involved in automated indexing in MEDLINE, and how has performance of the MTI evolved over time?
- **RQ3: Impact of automated indexing on searching:** What is the impact of automated indexing on MEDLINE searching, including effects on search filters, clinical queries, and comprehensive searches for knowledge synthesis?
- **RQ4: Stakeholders’ perceptions and responses:** How have key stakeholders (e.g., clinicians, librarians, indexers) perceived the changes due to automated indexing in MEDLINE, what concerns have they expressed, and how have they responded? What actions have they taken or integrated into their practices?

### Methods

Our scoping review methods were informed by Arksey and O’Malley’s 2005 framework [11], Levac et al’s 2010 update [12] and the JBI Manual for Evidence Synthesis (Scoping Reviews chapter) [13]. We developed an a priori protocol and shared it publicly via the Open Science Framework (OSF) in January 2025 [14]. We report our searches using PRISMA-S [15] and our review using PRISMA ScR [16].

### Data Sources and Literature Searching

We identified publications through multiple electronic search strategies. On 15 January 2025, we searched MEDLINE (Ovid), Embase (Ovid), CINAHL, Library Information Science and Technology Abstracts (LISTA) via EBSCO, Library and Information Science Abstracts LISA (ProQuest), Scopus, and Web of Science Core Collection (seven files: 1) Science Citation Index Expanded, 2) Social Sciences Citation Index, 3) Arts and Humanities Citation Index, 4) Conference Proceedings Citation Index, 5) Science, Conference Proceedings Citation Index, 6) Social Sciences and Humanities and 7) Emerging Sources Citation Index) [14].

To locate grey literature, we searched Figshare, Zenodo, ArXiv and medRxiv, the Open Science Framework (OSF), the NLM Technical Bulletins, available transcripts of PubMed Office Hours and the website of NLM’s Lister Hill National Center for Biomedical Communications (LHNCBC). We set up and received weekly alerts from 15 January 2025 to 15 October 2025 in Medline (Ovid), Embase (Ovid), LISTA (EBSCO) and the Web of Science (WoS) [14].

### Search Strategy Development

We developed two main concept blocks for our MEDLINE searches, which were combined using Boolean operators AND OR. No limits were applied for study design, language, or publication date [14]. The strategy was translated for use in the other biomedical and library science databases as specified in our protocol, and appropriate controlled terms were identified as needed. All search strategies and database strings, including details of our grey literature searching, are reported in Appendix B.

To increase the likelihood of identifying relevant publications not captured through database searching, we conducted targeted searching of the electronic table of contents of seven (7) key journals in health sciences librarianship (2000–2025): the *Journal of the European Association for Health Information and Libraries, Evidence Based Library and Information Practice, Journal of the Canadian Health Libraries Association, Journal of the Medical Library Association, Journal of eScience Librarianship, Medical Reference Services Quarterly, and Hypothesis.* These journals were selected based on their relevance to the field and their likelihood of publishing on MEDLINE indexing and its effects on information retrieval. Targeted searching of key journals is particularly important in this domain of health sciences librarianship where inconsistent indexing, evolving terminology, and delays in database inclusion may limit retrieval through standard search strategies. (For specific dates, volumes, and issues searched, see Appendix B.)

To ensure we did not miss any unpublished research, we searched all available electronic conference proceedings at three health sciences libraries’ websites: the Canadian Health Libraries Association (CHLA/ABSC), European Association for Health Information and Libraries (EAHIL), and the Medical Library Association (MLA). (For the specific conference proceedings websites, and dates searched, see Appendix B.)

### Reference Harvesting

We supplemented our bibliographic database search by reference harvesting. We identified seed papers (n=35) using MEDLINE (Ovid), Google Scholar and Web of Science [14], and searched their references for additional relevant publications. When locating relevant material, we added the full-text into our grey literature folder for eventual loading into Endnote and Covidence.

### Citation Management

We performed initial deduplication of records in Endnote using the Bramer method [17], then imported records into Covidence for additional deduplication and to begin the screening process. Zotero [18] was used for overall citation management and to format references in the manuscript.

### Inclusion and Exclusion Criteria

Two reviewers independently screened titles and abstracts against inclusion criteria, with discrepancies resolved through discussion. We considered all data sources (peer-reviewed and non-peer-reviewed) and methods (case reports, mixed methods, qualitative, and quantitative). We included publications when they addressed the following:

1. Drivers, benefits or challenges of automated indexing in MEDLINE;
2. Evolution of the Medical Text Indexer, or MTI, at NLM from 2000–2025;
3. Impact of automated indexing on accuracy, completeness and quality of records;
4. Librarians and researchers’ perspectives of automated indexing and the MTI.

We excluded publications published before 2000, or that did not discuss automated indexing using the MTI.

### Selection and Screening of Publications

#### Stage 1: Initial screening and deduplication

After deduplication in EndNote, citations were imported into Covidence, where any remaining duplicates were removed. Two pairs of reviewers independently screened the records using predetermined inclusion criteria. Screening was not blinded to author or journal information. Most titles and abstracts were in English; for two non-English publications, two team members with the requisite language skills (French and Japanese) translated the content to assess their eligibility. Any disagreements were resolved through discussion at regular team meetings until consensus was reached.

#### Stage 2: Full-text screening

Publications selected for full-text review were uploaded to Covidence. Any disagreements were resolved through team discussion, with all decisions documented throughout the process.

### Data Extraction

A standardized data extraction form was developed and pilot-tested in Google Sheets to ensure consistency and completeness. The form was iteratively refined following the pilot phase to ensure that all variables relevant to analysis were captured in a structured and reproducible way. The final form included fields for full citation details, country of origin, publication type, study aims and methods, the specific version of MTI under evaluation, and how each publication addressed each of the review questions (RQs).

For each variable, multiple data points were captured rather than any single summary judgment. Reviewers recorded descriptive information (e.g., MTI version, year of publication, study design), along with qualitative and quantitative findings related to indexing performance, errors, and evaluation methods. Structured fields and open-text fields were also used to capture key findings, reported outcomes, and noteworthy aspects of each publication, including any narratives about the underlying computer technologies evaluated (e.g., rule-based, machine learning, or hybrid approaches).

Data extraction was conducted independently by two reviewers for all included studies to enhance reliability. Discrepancies between reviewers were documented and resolved through discussion with a third reviewer and, when necessary, consultation with the entire team to reach consensus. This process resulted in a set of agreed-upon data points for each study. All extracted data were then compiled into a master spreadsheet, which enabled both descriptive analysis and thematic synthesis across studies.

### Thematic Analysis and Categorization

We identified publications and examined them using the thematic analysis techniques by Braun and Clarke [19, 20], supplemented with further guidance from the framework by Thomas and Harden [21]. For each publication, we examined how the four research questions were addressed, documented the specific version of MTI evaluated based on the year of publication or the authors explicitly mentioning the version under evaluation.

The publications were categorized by publication type and study method using MeSH publication types (e.g., “Journal Article,” “Conference Paper”) and study design terms (e.g., “Comparative Study,” “Evaluation Study”). Two additional categories - “Mixed Methods” and “Qualitative Descriptions” - were applied based on definitions from Sandelowski [22] and Schoonenboom et al. [23] (Appendix C).

### Results

**Figure 1:**
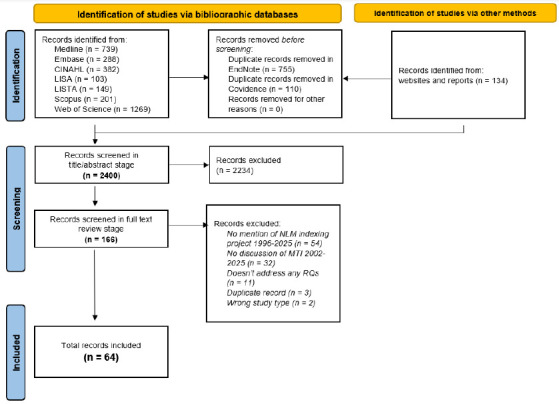
PRISMA-ScR flow diagram

Our searches yielded a total of 3,265 records from all searching in the bibliographic databases, journals, grey literature, and website searches, including reference harvesting and citation tracking. After removing 755 duplicates in EndNote and Covidence (which identified an additional 110 duplicates), 2,400 records remained for title and abstract screening. Of these, 2,234 were excluded, leaving 166 full-text publications assessed for eligibility. Following full-text review, 102 were excluded, resulting in 64 publications that met our inclusion criteria [9, 10, 24–85] (See Figure 1).

Summaries of our 64 publications and their key findings are presented in the table in Appendix H [9, 10, 24–85].

### Language and Publication Years

**Figure 2:**
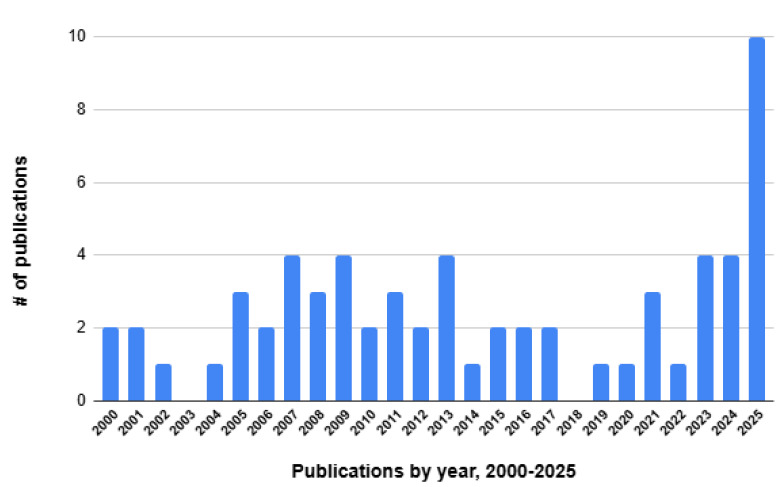
Publications by Year, 2000–2025

All 64 publications were published in English and spanned a 25-year period from 2000 to 2025 (see Figure 2). They were distributed as follows: 2000–2009 (n = 22, 34%), 2010–2019 (n = 19, 30%), and 2020–2025 (n = 23, 36%). Volume peaked in 2025 with ten publications (16%).

### Geographic Distribution

The US accounted for 83% of the included publications (n = 53) with NLM researchers contributing two-thirds of the total number (n = 42, 66%). NLM affiliation was determined by examining the institutional affiliations of first authors and recording this information in our data extraction form. First authors were classified as NLM-affiliated when their listed institution explicitly referenced NLM or one of its informatics or indexing divisions (e.g., NLM Index Section or LHNCBC). Full details are provided in Appendix D.

Canadian researchers contributed five publications (n = 5, 8%), examining topics such as MTI’s impact on check tags, search filters, and the indexing of minority groups. Nearly a dozen publications (n = 11, 17%) were published outside the US. In Europe, two publications originated from Portugal (n = 2, 3%) and one from Spain, with single publications from the United Kingdom and Denmark. One additional publication was conducted in Brazil.

### Publication Types

Conference papers comprised 44% of publications, followed by journal articles at 41%. Together, these formats accounted for 85% of research outputs (Table 1).

**Table 1:** Distribution of Publication Types

Publication Type	Count	Percentage
Conference Paper	28	44%
Journal Article	26	41%
Report	6	9%
Preprint	2	3%
Poster	1	2%
Commentary	1	2%
Total	64	

Six NLM publications (9%) provided authoritative updates on MTI enhancements, including implementation of the MTIA in 2022 and MTIX in 2024. Two publications (3%), retrieved from arXiv, reflected emerging trends in open science and rapid dissemination of machine learning research. One poster published a checklist for authors to ensure accurate indexing in the algorithmic era [24]. A single commentary by Neveol and colleagues [64] evaluated previous research by Trieschnigg et al. [66], noting that experiments with a ‘Nearest Neighbor’ algorithm were not reproducible, and that their findings did not align with previously published work about automated indexing.

### Journal Outlets and Conferences

Publications were from 16 journal outlets in total (Appendix E). The leading outlet was the *Journal of the Medical Library Association* (n = 4, 6%), followed by three each (5%) in *BMC Bioinformatics, Journal of Biomedical Informatics,* and *Research in Social and Administrative Pharmacy*. *Bioinformatics* and *JAMIA Open* each published two (3%). The remaining 10 journals span a diverse range of fields, including bioinformatics, library and information science, and the health professions. The American Medical Informatics Association (AMIA) conferences accounted for 18 publications (28%), while CEUR Proceedings Workshops contributed two (3%) and the Association for Information Science and Technology (ASIS&T) contributed one (2%). Six publications (9%) originated from the Biomedical Semantic Indexing and Question Answering (BioASQ) competition in 2013 [86].

### Study Methods

We classified publications into four methodological categories. A majority of publications (n = 34, 53%) met the MeSH definition of an evaluation study, examining the effectiveness or utility of processes, personnel, or systems. Sixteen (25%) were qualitative descriptions. Comparative studies (n = 7, 11%) assessed outcomes across different techniques or approaches, while seven (11%) employed mixed methods (Appendix E).

## DISCUSSION

### Thematic Analysis

**Figure 3:**
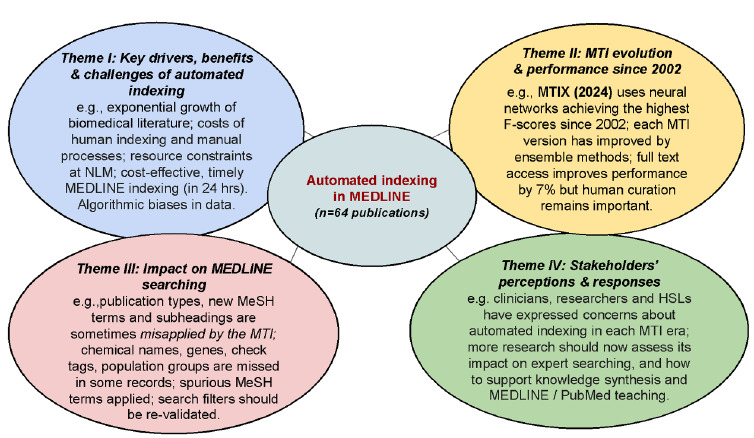
Key Themes Based on Four (4) Research Questions

We organized our findings into four analytical themes, namely: (1) key drivers, benefits, and challenges of automated indexing; (2) the evolution and performance of the MTI since 2002; (3) impact on MEDLINE searching; and (4) stakeholders’ perceptions and responses to automated indexing in MEDLINE. We summarize key points in Figure 3.

### Theme I: Key Drivers, Benefits and Challenges of Automated Indexing

#### Drivers

Key drivers toward automated indexing included the exponential growth of publications in biomedicine [62, 74], increased volume of articles requiring MEDLINE indexing [26, 79], and the substantial investments - particularly in training and retraining indexers - needed to perform traditional manual indexing [25, 28, 35, 83].

#### Benefits and Challenges

Two key benefits of automated indexing were reduced indexing time and lower administrative costs [34, 65, 82]. Several publications highlighted indexing backlogs and dwindling resources at NLM as drivers of automation [52, 68], noting that human indexing was often slow and labor-intensive, even with considerable expertise, and unable to keep pace with rising indexing demands [25, 40, 46, 58]. The urgent need to implement automated methods was further illustrated by the backlogs and the growing number of new MEDLINE records, reaching 1.4 million in 2022 [4, 35].

Multiple publications reported concerns about the accuracy, consistency, and completeness of records [36, 55, 77], precision errors [38, 68, 76], and missed concepts or inappropriate check tags [26, 37]. Other challenges with automated indexing were MTI’s difficulty in interpreting figurative language and its misinterpretation of metaphors in titles and abstracts [10]. For example, the MTIX incorrectly assigned the MeSH term Malus (apple) to 135 of 1,705 records (8%) in which “apple” was used figuratively or as part of a name or term [33].

Early MTI testing in 2002 identified limitations in handling acronyms [60, 81], abbreviations, chemical and gene names [71], and numerical expressions [81]. Although NLM has since refined the MTI to improve the indexing of genes, proteins, and frequently searched topics in PubMed [9], these developments have not fully resolved underlying issues. By 2023, HSLs reported that the MTI perpetuated gender and equity biases, including a tendency to favor the male check tag over the female [37].

Several publications demonstrated that indexing outputs based on a publication’s full text, or portions thereof, improved MTI’s overall F-scores [54, 76]. F-scores represent the weighted mean of precision and recall and are expressed as a single numerical value or percentage [39]. However, NLM does not maintain subscriptions to all journals indexed in MEDLINE, and not all MEDLINE journals permit text mining of full text [73].

### Theme II: Evolution and Performance of the MTI

**Figure 4:**
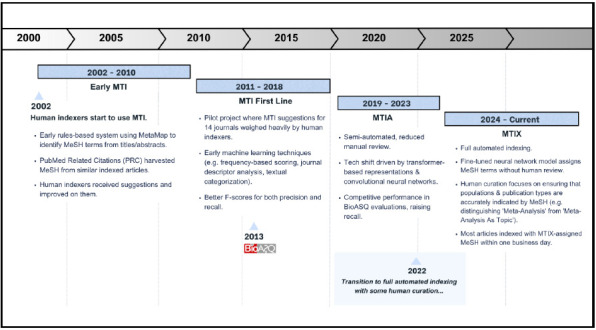
Timeline of MTI’s Evolution from 2002-Present

Several key publications traced the development and evolution of automated indexing in MEDLINE highlighting numerous key enhancements to the MTI (2002): 2011 (MTIFL), 2019 (MTIA), and 2024 (MTIX). A timeline of MTI’s evolution is shown in Figure 4.

Across publications, new technological innovations were tested by NLM, iterating and improving the rules-based, deterministic system of 2002 [63] to the neural network algorithm of 2024 [10]. Rules-based approaches between 2002 and 2022 were error-prone and required human intervention [37, 73], often missing synonyms and struggling with emerging terminology [42]. MTIA projects reported between 2010 to 2020 explored ensemble approaches to improve indexing outputs [43, 72], combining dictionary-based rules, natural language processing, and supervised learning. Ensemble methods were shown to consistently outperform single-approach systems [41].

#### Early MTI Development (2002–2010)

The first MTI had two core components: MetaMap and PubMed Related Citations (PRC) [79]. MetaMap identified concepts in text by mapping them lexically to the Unified Medical Language System (UMLS) [81, 82]. UMLS then mapped text to closely related MeSH terms using Restrict to MeSH [79], an algorithm that identified semantic relationships between concepts. To support MetaMap, PRC searched for existing indexed records similar in content to the item being indexed, based on the nearest neighbors (k-NN) algorithm [56, 81]. MTI integrated outputs from both components, extracted candidate MeSH terms, and applied weighting and post-processing to generate final indexing recommendations. NLM indexers had the option to use MTI recommendations or not based on their judgment; in fact, some experienced indexers ignored them altogether or used them to ensure they didn’t miss major topics [46].

#### MTI First Line (2011–2018)

In 2011, MTIFL became the first-line indexer for 14 journals, expanding to 51 by 2015 [39, 47, 49]. MTIFL nearly doubled its precision of MeSH recommendations for human indexers by using new precision and recall filters.

The MTIFL incorporated Journal Descriptor Indexing (JDI) and journal subsets to assign weights to candidate MeSH terms [63, 74, 85]. Bayesian classifiers trained on approximately four million MEDLINE citations were used to predict 127 discipline categories (e.g. Cardiology, Medical Genetics) [74]. By leveraging the statistical associations among journal-level metadata, textual features, and MeSH terms, JDI achieved high levels of precision (82–97%) and recall (75–92%). Improvements to the MTIFL marked the first of many small steps toward fully automated indexing [39].

#### MTI Auto (2019–2023)

In 2019, NLM implemented a pilot of the MTIA starting with some journals, which marked a major step towards fully automated indexing [9, 40, 58]. MTIA’s development drew on lessons learned from the BioASQ Semantic Indexing Challenges and refinements from two short-lived initiatives, MTI Review (MTIR) and MTI Comment On (MTIC) [39]. By 2021, NLM had expanded the MTIA to cover 40% of journals indexed in MEDLINE, and by 2022 it was central to the transition to fully automated indexing [9, 39].

By 2023, processing times had dropped from months to days, and the MTIA was handling most routine MEDLINE indexing [39]. NLM’s adoption of deep learning yielded further performance gains, with F-scores rising from approximately 0.40–0.50 to 0.65–0.75 [9, 10]. Together, these advances in efficiency and scalability enabled NLM to manage indexing workloads that would otherwise have been unsustainable [9, 10].

#### MTIX: Neural Networks – Transformer Models (2024-Present)

By 2024, NLM had fully transitioned to the neural network–based MTIX, representing state-of-the-art automated indexing using artificial intelligence (AI). Formal testing of convolutional neural network (CNN) models began in 2012 and continued for a decade [41, 42, 45]. Unlike early MTI systems relying on fixed dictionaries, trigger phrases, and human-crafted rules, MTIX used AI-based neural networks as its main technology [10].

MTIX deployment improved performance for publication types and check tags, two categories with high search impact in MEDLINE [10]. Human curation continues for roughly one-third of all MTIX-indexed articles, focusing on high-impact journals, specialized publication types, and complex or high-demand subjects [10]. MTIX is a machine learning algorithm and needs human curation to learn about any new MeSH terms [10]. Most articles indexed in MEDLINE now appear in PubMed with MTIX-assigned MeSH terms within one business day [4].

**Figure 5:**
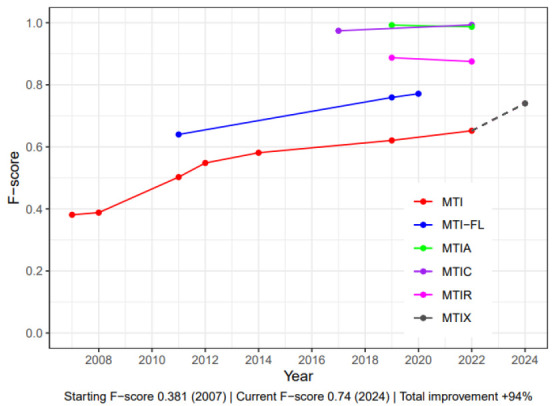
Performance of MTI (2002) to MTIX (2024)

Performance evaluations of the MTI algorithms from 2002 to 2025 demonstrate substantial improvement across standard metrics [27]. In several publications, F-scores were reported to have increased from 0.38 in 2007 to 0.74 in 2024 [10, 27, 39]. Early evaluations reported modest performance (F ≈ 0.38–0.58), reflecting a limited balance between sensitivity and specificity, whereas later versions incorporated filtering, ranking, and neural methods that contributed to marked gains for a total improvement of 94%. (MTI’s performance and F-scores from 2007 to 2024 are in Figure 5.)

#### Deep Learning and Transformer-Based Models

From 2020–2025, NLM continued to integrate deep learning and transformer-based language models to improve automated indexing processes [27, 31, 35]. BERT (Bidirectional Encoder Representations from Transformers) is a neural network model designed for natural language processing, enabling machines to understand text with a human-like contextual awareness [39, 42, 44].

More specific transformer models such as DistilBERT, BioBERT, PubMedBERT, and SciBERT demonstrated strong performance on complex NLP tasks and MeSH prediction, paving the way for more highly scalable, efficient MEDLINE indexing [27, 44]. (See Appendix G for more details on BERT models' features, and their environmental footprint.)

### Theme III: Impact on MEDLINE Searching

Numerous publications highlighted concerns for MEDLINE searchers, noting several discipline-specific difficulties due to automated indexing and the impact of indexing errors and under-indexing of certain populations and subjects.

#### Implications for Health Sciences Librarians (HSLs)

Several publications discussed the impact of errors on precision and recall, search filters, and publication types [25, 31, 36, 69, 81] with explicit recommendations to exercise caution in creating search strategies [25, 37]. In light of emerging errors in MEDLINE, HSLs suggested revalidating (or at least testing) previously validated search filters [25].

One publication suggested that HSLs assess changes to MTI-assigned terms, and their impact on mapping features for various topics and interfaces [37]. Another advised HSLs to test subheadings, broader terms in hierarchical trees, and add more free text terms to compensate for missing or ambiguous indexing [25]. Finally, two publications recommended that researchers wanting to improve their articles' discoverability should ensure key concepts are mentioned explicitly in titles and abstracts, even using terminology matching existing MeSH terms [24, 25].

Across publications, several recurring recommendations were identified for both HSLs and researchers. For example, HSLs should critically assess changes to MTI-assigned terms and evaluate their impact on mapping features across topics and MEDLINE search interfaces [37]. In response to limitations in automated indexing, HSLs also recommended the testing of subheadings, exploring broader terms within hierarchical structures, and supplementing searches with additional free-text terms to mitigate missing or ambiguous indexing [25].

In parallel, several publications highlighted the role of authors in improving discoverability, noting that explicitly stating key concepts in titles and abstracts using terminology aligned with MeSH terms will enhance later indexing and allow the MTI to assign them more readily [24, 25]. These findings reflect that, despite advances in automated indexing, searchers and authors are already actively compensating for its limitations.

#### Specific Challenges in Pharmacy, Genetics and Chemistry

In three publications, pharmacists reported that their literature was poorly indexed and missed concepts consistently [29, 34, 36]. Researchers noted that the MeSH thesaurus included only 26 pharmacy-specific terms compared to 94 terms for nursing and 145 for dentistry which also contributed to weaker indexing and retrieval outcomes [50].

In genetics and gene-related indexing, specialized approaches were also required [71]. One publication revealed that domain-specific document representations (incorporating gene names and sequence information) produced higher F-scores than generic text-based representations, though performance remained limited in testing [71]. Deep learning models further improved results, with BERT fine-tuning outperforming earlier approaches for gene named entity recognition, while gene normalization to Entrez Gene identifiers remained comparatively more challenging. [31, 71].

Chemical entity recognition similarly required specialized approaches [44]. Evaluation of 11 chemical recognition systems on 200 annotated MEDLINE abstracts found that the SciBERT-Ensemble achieved the highest F-score, while MTI scored the lowest [44]. Since the MTI was not designed for chemical recognition, external chemical entity extractors were needed for adequate performance in chemistry-focused articles [44].

#### Indexing Errors and Under-Indexing of Certain Populations and Topics

A handful of publications revealed that certain populations and topics were affected by algorithmic indexing errors and under-indexing. Research concerning intersex populations, for example, was shown to be poorly indexed, reflecting gaps and inconsistencies in how such topics are represented in MEDLINE [32]. A newer MeSH term, Overdiagnosis, was shown to be misapplied at times [30], and inaccuracies were observed in indexing patient simulation literature [36]. Across subject areas, publications in pharmacy [29, 34], genetics [71], and allied health [37] were more likely to have fewer, missed, or misassigned MeSH terms. These inconsistencies in MeSH indexing are revealing case studies and reflect and reinforce underlying biases in algorithmic indexing practices noted by Chen et al [37].

When comparing automated indexing of human and animal studies, MTIX-indexed records demonstrated significantly greater accuracy than those records indexed by the MTIA [28]. Consistent with these findings, several publications caution against uncritical application of search filters: for example, the Cochrane Human filter may perform less reliably for records indexed between 2019 and 2024 [26].

Taken together, the evidence highlights systematic gaps in automated indexing across certain populations, topics, and disciplines, underscoring the need for careful evaluation and, where appropriate, supplementary search strategies. NLM’s reliance on statistical approaches, and subsequent expansion into machine learning, has contributed to inheriting and amplifying existing problems and biases in the MTI [37].

To mitigate this persistent issue, increased human curation will be necessary for publications about or involving underrepresented populations and rapidly evolving concepts known to pose challenges for the MTI [33, 37]. Ongoing feedback from HSLs can contribute to a better calibrated system by reporting indexing errors and biases [37], including MeSH assignments that are problematic, harmful, or discriminatory.

### Theme IV: Stakeholders’ Perceptions and Responses

In this theme, multiple stakeholders – such as authors, indexers, librarians, pharmacists, and NLM curators and researchers - expressed concerns about automated MEDLINE indexing, particularly its impact on the accuracy, consistency, and completeness of records. Table 2 summarizes their perceptions and how they responded through changes in their practices. We provide more detail about the perceptions of two stakeholder groups internal to the NLM that were not addressed in previous themes: NLM indexers and curators, and NLM researchers and staff scientists.

**Table 2:** External / internal stakeholders’ key perceptions and responses

External Stakeholders	Key perceptions & responses	Internal Stakeholders	Key perceptions & responses
**Authors & Editors** [24,29,36]	Important concepts should be included in titles/abstracts to assist indexing algorithm Authors should use words in titles/abstracts that match existing MeSH terms Clearly define populations in abstracts Use structured abstracts when possible	**NLM Indexers** [47,65,73,79]	NLM indexers said recommendations were too general; important MeSH were missing Less-experienced indexers relied on MTI but had concerns re: vague or mismatched entries Indexers valued the MTI, even though confidence and quality concerns persisted
**Health Sciences Librarians (HSLs)** [24,25,26,32,33,37]	HSLs suggest adding free text terms to searches to compensate for MeSH errors Broader MeSH are recommended when specific terms are missing/incomplete Previously validated search filters may need revalidation due to indexing changes	**NLM Curators** [31,39]	Curators identify ambiguity in gene nomenclature as persistent indexing challenge Articles requiring curation or human intervention about one third of MTIX-indexed content is an ongoing challenge Curators recognize that past human indexing decisions are valuable assets for training and improving algorithms
**Pharmacists** [29,34,36,50]	Gaps in pharmacy-related MeSH terms affect discoverability of pharmacy research Pharmacists express concern about poor indexing, and want to work with librarians Poor indexing also affects consistent retrieval in MEDLINE	**NLM Researchers / Staff Scientists** [31,39,44,48]	Automated chemical entity and gene linking in PubMed remains a work in progress Domain-specific representations are needed for accurate chemical entity indexing Researchers continue to explore fine-tuning approaches (e.g., BERT models) and ensemble methods to improve indexing performance

#### NLM Indexers and Curators

This category includes NLM indexers and curators described as such in included publications; these terms were used interchangeably, and sometimes as distinct descriptors. In several publications, “indexers” refer to professionals responsible for assigning controlled vocabulary terms (e.g., MeSH) to MEDLINE records, whereas “curators” denoted subject specialists who refined algorithms, resolved ambiguities, or addressed domain-specific data quality issues, such as gene or genomic indexing. This conceptual overlap, combined with inconsistent terminology across publications, highlighted the need for clearer role definitions within NLM and in the research literature. Greater conceptual clarity would also strengthen the interpretation of stakeholder perspectives and improve assessment of the perceived impact of automated indexing.

From the outset, NLM’s in-house and contract indexers have provided valuable insights into MTI’s performance. In 2004, 37% of indexers described MTI as “fully helpful,” and 53% as “partially helpful” for assigning MeSH terms to records, while 20% expressed some concerns about mismatched entries and vague terms [79]. By 2007, less-experienced indexers appeared to rely heavily on MTI [73]. Overall, 70–80% of MTI’s top 10 recommendations were utilized by all human indexers [47].

In 2025, specialized indexers, referred to as “curators,” were surveyed to inform NLM’s efforts to improve gene linking from PubMed publications [31]. These curators identified persistent challenges, including textual ambiguity in gene nomenclature and the rapid growth of genomic research during and following the COVID-19 pandemic. Their feedback contributed to enhancements of the GNorm2 algorithm, expanding its coverage to include genes from 135 viral and bacterial species [31].

#### NLM Researchers and Staff Scientists

Researchers and staff scientists at NLM’s LHNCBC have conducted research to address main heading / subheading attachment problems of the MTI [41, 65, 72], incomplete indexing of study populations and publication types [9, 27, 54, 80], and the use of full text to improve performance [49, 52, 53]. Informatics researchers continue to examine the value of crawling the full text, although full-text documents are not always accessible to the MTI [35]. In one publication, automated summaries were used in the MTI as alternatives to full text, but this approach was never integrated [49].

In 2012, NLM scientists recognized that no single automated indexing system performed optimally across all document types and domains [56]. Subsequently, MTI performance was enhanced using a meta-learning framework which combined multiple indexing methods to address challenges such as limited datasets and infrequently used MeSH terms [56]. In publications from 2019 to 2023, staff scientists confirmed the value of combined approaches in achieving high-quality MeSH indexing and improving F-scores [41, 44, 45].

### Research Gaps and Future Directions

In this review, we identified only one publication that examined indexing quality in a foreign language (French) [78], despite MEDLINE’s inclusion of articles published in nearly 40 languages. Examinations of indexing quality were concentrated in pharmacy, genetics, and public health, and we noted that many medical specialties have not yet conducted discipline-specific evaluations. Investigating mis-indexed records related to racialized and other marginalized groups seems to be a major gap and urgent priority, particularly where outdated, problematic, or racist terminology has been applied [88].

Further research is needed on the temporal dimension of automated indexing and its compounding downstream effects on MEDLINE search filters. Prior to automated indexing, inter-indexer inconsistency posed persistent challenges to quality control [89]. As an increasing proportion of MEDLINE records are now indexed algorithmically, the performance of existing search filters may require systematic revalidation.

To date, no publications have assessed the carbon emissions, energy consumption, water usage or labor impact associated with training or deploying AI-based indexing systems. Given the substantial computational resources required to operate AI technologies [90], future research should evaluate MTIX’s environmental footprint alongside traditional performance metrics. Additionally, qualitative research involving HSLs and other expert searchers are needed to better understand how AI-based indexing is reshaping MEDLINE search strategies and PubMed instruction. Findings from this work could inform training programs and support the development of best practices for HSLs.

### Limitations

This review has some limitations. Despite the number of sources searched, some perspectives may have been overlooked, and we may have missed research that focused on MeSH indexing but did not explicitly mention the MTI. The highly technical nature of some of the computer science and informatics papers presented challenges for us; while we shared our understanding with each other at meetings, created a glossary of terms and a wiki to deepen our machine learning literacies [4], these publications would benefit from closer analysis by domain experts.

We did not assess the quality of publications using formal critical appraisal tools. Although consistent with standard scoping review methods, this limited our ability to make definitive statements about the strength of evidence. Our predominant inclusion of English-language sources, despite including publications that could be translated, may have introduced language bias.

### Conclusion

The transition to automated indexing represents one of the most significant changes to MEDLINE in NLM’s 150-year history. While improvements to the MTI from 2000 to 2025 have been transformative, automated indexing systems have not yet achieved full equivalence with human indexing. HSLs will continue to play critical roles in the future of algorithmic indexing, particularly in evaluation, advocacy, and ongoing oversight. Future HSL-led research should focus on supporting evidence synthesis, advancing effective MEDLINE teaching practices, and addressing mis-indexed articles affecting marginalized populations due to algorithmic errors and biases.

## SUPPLEMENTAL FILES

**Appendix A:** Glossary of Terms and Abbreviations

**Appendix B:** Search Strategy Development

**Appendix C:** Definition(s) of Study Methods

**Appendix D:** Most Cited NLM and Other Researchers (2000–2025)

**Appendix E:** Publication Venue

**Appendix F:** Study Methods Distribution

**Appendix G:** BERT-Based Models

**Appendix H:** Table: Summaries of 64 Publications
