# Supplementary material for: Automated indexing in MEDLINE and the Medical Text Indexer (MTI), 2000–2025: a scoping review
Source: J Med Libr Assoc. 2026 Jul 14;114(3):191–207. doi: 10.5195/jmla.2026.2406 (PMC13367316; doi:10.5195/jmla.2026.2406)
Supplement: Supplementary file 1 — Appendix A: Glossary of Terms and Abbreviations [file jmla-114-3-191-s01.pdf]

Appendix A: Glossary of terms and abbreviations

- AI (Artificial Intelligence) – Computer systems performing tasks such as learning, prediction, and pattern recognition
- Automated (or algorithmic) indexing – form of text categorization and pattern recognition where computers use algorithms and natural language processing (NLP) to assign controlled terms (like MeSH) to documents in a database summarizing their content for comprehensive information retrieval
- Bayesian classifier – a probabilistic model used to facilitate automatic text indexing, and backbone of the early MTI.
- BERT (Bidirectional Encoder Representations from Transformers) – language model used to improve indexing, classification, and textual understanding.
    - BioBERT is pre-trained on biomedical text: PubMed and PMC
    - DistilBERT is a smaller, faster, and lighter version of original BERT PubMedBERT is a transformer model pretrained on PubMed text SciBERT is a model pre-trained on biomedical and computer science papers from Semantic Scholar
- BioASQ (Biomedical Semantic Indexing and Question Answering challenge) – annual competition event benchmarking large-scale MeSH indexing and biomedical question answering systems, sponsored by NLM among others
- Check tags – specific Medical Subject Headings (MeSH) routinely assigned to articles indexed in MEDLINE to indicate fundamental study characteristics, such as species, sex, age groups, and publication types. Examples include Humans, Animals, Male, Female, Child, Adult, Aged, and publication types such as Randomized Controlled Trial or Review.
- CNN (Convolutional Neural Network) – deep-learning architecture used for automated MeSH indexing and document classification used in the MTIX
- COORD (Indexing Coordination Rules) – rules for combining MeSH terms to reflect complex, coordinated subject headings
- DCMS (Data Creation and Maintenance System) – NLM interface where indexers view and edit MeSH term suggestions
- DeepMeSH – deep semantic representations of text to capture context and meaning, ranking MeSH terms from large datasets
- EAGL (Example-based Automatic General Labeler) – early system using examples to assign MeSH-like subject labels
- F-score (F1-measure) – harmonic mean of precision and recall, summarizing overall indexing performance in a single metric
- IAD (Indexer Assignment Dataset) – large dataset to assist assigning relevant articles to well-trained human indexers
- IND – Indexing Initiative of the NLM, dating back to the 1990s
- JDI (Journal Descriptor Indexing) – classifier assigning broad subject labels to articles, supporting MeSH term ranking
- k-NN (k-Nearest Neighbors) – method that uses similar indexed articles to recommend most likely related terms and citations

- LTR (Learning to Rank) – machine-learning approach that orders MeSH terms by their predicted relevance to a given text
- MAIF – MAIF (MeSH Automatic Indexer for French) refers to an automated system for indexing French-language biomedical texts, often developed within projects like VUMeF (French Unified Medical Language System, part of the larger UMLS), which uses Natural Language Processing (NLP) and knowledge-based methods to assign MeSH (Medical Subject Headings) terms similar to NLM's MTI but specialized for French resources.
- MeSH (Medical Subject Headings) – controlled vocabulary used by MEDLINE to index biomedical articles
- MetaMap – NLM tool mapping free-text to UMLS biomedical concepts, an NLM resource linking many medical vocabularies
- MTI (Medical Text Indexer) – NLM's original 2002 tool suggesting MeSH terms for (semi)automated MEDLINE indexing
- MTIA (Medical Text Indexer – Automated) – fully automated 2023 MTI mode assigning MeSH without routine human review
- MTIC – MTI Comment On started in 2017 and was a short-lived project. It looked at the title of the article being commented on to enrich the text of articles commenting on other articles. MTIC replaced the practice of just copying over indexing from the originating article.
- MTIFL (Medical Text Indexer – First Line) – a 2011 workflow where MTI suggestions are applied then examined by indexers
- MTIR – MTI Review was used by NLM from 2017 to 2022 and was a short-lived project. It examined how journals performed using the MTIA algorithm. Indexers would review every article indexed by MTIR only using the title and abstract (as opposed to the full text of an article which they normally indexed from) to evaluate MTIR indexing.
- MTIX (Medical Text Indexer – neural network-based) – introduced in 2024, MTIX is an AI-based automated indexing tool; reduces errors with improved scalability
- NLM (National Library of Medicine) – US library creating MEDLINE and developing MTI-based indexing systems to support it
- Naive Bayes classifier – probabilistic model used to facilitate automatic text indexing, and the backbone of early MTI
- NLP (Natural Language Processing) – early techniques for analyzing biomedical texts in order to recognize concepts and assign MeSH terms automatically
- NN (Neural Network) – computational model of layered nodes of learned patterns for classification and prediction purposes
- PRC (PubMed Related Citations) – uses citation and content similarity to suggest MeSH terms based on similar articles.
- SVM (Support Vector Machine) is a powerful supervised machine learning algorithm used for both classification and regression tasks, and finds an optimal decision boundary that maximally separates important data points belonging to different classes; SVM draws the single most robust, widest possible boundary that focuses only on the examples that actually matter.
- UMLS (Unified Medical Language System) – NLM resource linking many vocabularies; MetaMap maps text to the UMLS;

- UMLS semantic types (STs) – are high-level categories UMLS uses to group similar concepts before mapping to MeSH terms
- Vector embedding – transforms unstructured data into structured format machine learning models can efficiently process and interpret. This allows machines to understand abstract concepts like semantic meaning and context
- WSD (Word Sense Disambiguation) – methods for choosing correct conceptual meaning, when terms or abbreviations are ambiguous
