# Supplementary material for: Automated indexing in MEDLINE and the Medical Text Indexer (MTI), 2000–2025: a scoping review
Source: J Med Libr Assoc. 2026 Jul 14;114(3):191–207. doi: 10.5195/jmla.2026.2406 (PMC13367316; doi:10.5195/jmla.2026.2406)
Supplement: Supplementary file 2 — Appendix B: Search Strategy Development [file jmla-114-3-191-s02.pdf]

Appendix B – Search strategies used in the bibliographic databases

MEDLINE (Ovid) <1946 to January 15th, 2025>

#	Query	Results
1	*"Abstracting and Indexing"/	2,526
2	((algorithm* or mechani* or automat* or computer*) adj5 (categor* or classifi* or index*)).ti,ab,kf.	37,482
3	(algorithm* adj3 index*).ti,ab,kf.	696
4	(automat* adj3 index*).ti,ab,kf.	875
5	(computer* adj3 index*).ti,ab,kf.	406
6	(human adj3 index*).ti,ab,kf.	3,582
7	(machine adj3 index*).ti,ab,kf.	198
8	(manual adj3 index*).ti,ab,kf.	251
9	(mechan* adj3 indexing).ti,ab,kf.	41
10	(medical adj3 index*).ti,ab,kf.	2,641
11	(MTI or MTIA or MTI-A or MTIFL or MTI-FL or MTIX or MTI-X).mp.	1,088
12	(semantic adj3 index*).ti,ab,kf.	317
13	"semi automated index*".ti,ab,kf.	2
14	"semi automatic index*".ti,ab,kf.	4
15	or/1-14	47,553
16	Medline/	5,207
17	exp Subject Headings/	1,545
18	(medical adj3 heading*).ti,ab,kf.	4,605
19	subject heading*.ti,kf.	260

20	medline.ti,kf.	1,204
21	exp PubMed/	8,027
22	pubmed.ti,kf.	1,400
23	"national library of medicine".ti,ab,kf.	3,542
24	NLM.ti,kf.	227
25	qualifier*.ti,ab,kf.	896
26	("subheading*" or "sub-heading*").ti,ab,kf.	773
27	or/16-24	17,382
28	15 and 27	739

Note: Search alerts in Medline (Ovid) were run weekly from January 15th to October 15th 2025 in order to keep this review current. As new papers were published, we added them to Covidence for screening.

EMBASE (Ovid) <1974 to 2025 January 15>

#	Query	Results
1	automat* index*.ti,ab,kf.	201
2	algorithm* index*.ti,ab,kf.	23
3	((algorithm* or automat* or computer* or mechani*) adj3 (categor* or classifi* or index*)).ti,ab,kf.	31,893
4	human index*.ti,ab,kf.	77
5	(machine adj3 index*).ti,ab,kf.	232
6	("mechanical indexing" or "mechanized indexing").ti,ab,kf.	3
7	(medical adj2 index*).ti,ab,kf.	2,608
8	medical text indexer.ti,ab,kf.	29

9	("medline index*" or "pubmed index*").ti,ab,kf.	916
10	(MTI or MTIA or MTI-A or MTIFL or MTI-FL or MTIX or MTI-X).ti,ab,kf.	1,447
11	(qualifier* adj10 (automat* or index*)).mp.	24
12	semantic index*.ti,ab,kf.	124
13	semi automated index*.mp.	2
14	"semi automatic index*".mp.	4
15	("subheading*" or "sub-heading*").ti,ab,kf. adj10 (automat* or index*).mp.	42
16	text* index*.ti,ab,kf.	230
17	or/1-16	37,374
18	exp *bibliographic database/	4,922
19	*medline/	2,009
20	medline.ti,kf.	1,559
21	*medical subject headings/	271
22	subject heading*.ti,kf.	289
23	pubmed.ti,kf.	1,464
24	"national library of medicine".ti,kf.	282
25	or/18-24	7,006
26	17 and 25	288

Note: Search alerts in Embase (Ovid) were run weekly from January 15th to October 15th 2025 in order to keep this review current. As new papers were published, we added them to Covidence for screening.

Search strategies in LISTA and CINAHL

COMMAND: TX (("automat* index*" OR "algorithm* index*" OR "computer* index*" OR “human index*” OR "machine index*" OR "mechanical indexing" OR "mechanized indexing" OR "medical index*" OR "medical text indexer" OR "medline index*" or "pubmed index*" OR MTIA or MTI-A or MTIFL or MTIX or MTI-X OR qualifier* OR “semantic index*” OR “semi automated index*” OR “subheading*”) AND (MEDLINE

OR PubMed OR "medical subject heading*" OR MeSH OR "national library of medicine" OR NLM))

N=149 Papers Found in *LISTA – *We also set an automatic alerting update in LISTA (EBSCO) and programmed it to run weekly from January 15th to October 15th 2025.

https://search.ebscohost.com/login.aspx?direct=true&AuthType=shib&db=lxh&bquery=TX+((%26quot%3bautomat*+index*%26quot%3b+OR+%26quot%3balgorithm*+index*%26quot%3b+OR+%26quot%3bcomputer*+index*%26quot%3b+OR+%e2%80%9chuman+index*%e2%80%9d+OR+%26quot%3bmachine+index*%26quot%3b+OR+%26quot%3bmechanical+indexing%26quot%3b+OR+%26quot%3bmechanized+indexing%26quot%3b+OR+%26quot%3bmedical+index*%26quot%3b+OR+%26quot%3bmedical+text+indexer%26quot%3b+OR+%26quot%3bmedline+index*%26quot%3b+or+%26quot%3bpubmed+index*%26quot%3b+OR+MTIA+or+MTI-A+or+MTIFL+or+MTIX+or+MTI-X+OR+qualifier*+OR+%e2%80%9csemantic+index*%e2%80%9d+OR+%e2%80%9csemi+automated+index*%e2%80%9d+OR+%e2%80%9csubheading*%e2%80%9d)+AND+(MEDLINE+OR+PubMed+OR+%26quot%3bmedical+subject+heading*%26quot%3b+OR+MeSH+OR+%26quot%3bnational+library+of+medicine%26quot%3b+OR+NLM))&type=1&searchMode=Standard&site=ehost-live&scope=site&custid=s5672194

N=382 Papers Found in CINAHL -
https://search.ebscohost.com/login.aspx?direct=true&AuthType=shib&db=ccm&bquery=((%26quot%3bautomat*+index*%26quot%3b+OR+%26quot%3balgorithm*+index*%26quot%3b+OR+%26quot%3bcomputer*+index*%26quot%3b+OR+%e2%80%9chuman+index*%e2%80%9d+OR+%26quot%3bmachine+index*%26quot%3b+OR+%26quot%3bmechanical+indexing%26quot%3b+OR+%26quot%3bmechanized+indexing%26quot%3b+OR+%26quot%3bmedical+index*%26quot%3b+OR+%26quot%3bmedical+text+indexer%26quot%3b+OR+%26quot%3bmedline+index*%26quot%3b+or+%26quot%3bpubmed+index*%26quot%3b+OR+MTIA+or+MTI-A+or+MTIFL+or+MTIX+or+MTI-X+OR+qualifier*+OR+%e2%80%9csemantic+index*%e2%80%9d+OR+%e2%80%9csemi+automated+index*%e2%80%9d+OR+%e2%80%9csubheading*%e2%80%9d)+AND+(MEDLINE+OR+PubMed+OR+%26quot%3bmedical+subject+heading*%26quot%3b+OR+MeSH+OR+%26quot%3bnational+library+of+medicine%26quot%3b+OR+NLM))&type=1&searchMode=Standard&site=ehost-live&scope=site&custid=s5672194

<u>Search strategies in LISA</u> N=103 Papers

<u>Use this search string and command:</u>

- noft((("automat* index*" OR "algorithm* index*" OR "computer* index*" OR “human index*” OR "machine index*" OR "mechanical indexing" OR "mechanized indexing" OR "medical index*" OR "medical text indexer" OR "medline index*" or "pubmed index*" OR MTIA or MTI-A or MTIFL or MTIX or MTI-X OR qualifier* OR “semantic index*” OR “semi automated index*” OR “subheading*”) AND (MEDLINE OR PubMed OR "medical subject heading*" OR MeSH OR "national library of medicine" OR NLM))

<u>Search strategies in citation indexes</u>

Web of Science Core Collection (all seven files) N= 1269 papers – *We also set an automatic alerting update in WoS and programmed it to run weekly from January 15th to October 15th, 2025.
https://www.webofscience.com/wos/woscc/summary/3ae01fe9-6be8-4b49-9c64-66df83ec72ea-0115616578/relevance/1

Searching in the topic field:

- (("automat* index*" OR "algorithm* index*" OR "computer* index*" OR “human index*” OR "machine index*" OR "mechanical indexing" OR "mechanized indexing" OR "medical index*" OR "medical text indexer" OR "medline index*" or "pubmed index*" OR MTIA or MTI-A or MTIFL or MTIX or MTI-X OR qualifier* OR “semantic index*” OR “semi automated index*” OR “subheading*”) AND (MEDLINE OR PubMed OR "medical subject heading*" OR MeSH OR "national library of medicine" OR NLM))

Search in Scopus (Elsevier) and alerts
N=201 Papers Found in Scopus- Searching in the title and keyword fields:

- (("automat* index*" OR "algorithm* index*" OR "computer* index*" OR “human index*” OR "machine index*" OR "mechanical indexing" OR "mechanized indexing" OR "medical index*" OR "medical text indexer" OR "medline index*" or "pubmed index*" OR MTIA or MTI-A or MTIFL or MTIX or MTI-X OR qualifier* OR “semantic index*” OR “semi automated index*” OR “subheading*”) AND (MEDLINE OR PubMed OR "medical subject heading*" OR MeSH OR "national library of medicine" OR NLM))

Search strategies used in supplemental and grey literature searching

We searched the following sources (pages 6–7) using the native search interfaces available for each resource, and documented our searches using a Google spreadsheet. Retrieved items were also saved to a shared Google Drive folder and subsequently imported into our citation management software for subsequent screening and analysis. Several of the websites searched supported browsing rather than structured searching; in those cases, we downloaded full-text documents and searched them electronically (e.g., using Ctrl+F) to identify relevant content. Where feasible, we used terms and phrases to locate items such as algorithmic indexing, automated indexing, automatic indexing, computerized indexing, human indexing, manual indexing, machine indexing, mechanized indexing, Medical Text Indexer, MEDLINE indexing, PubMed indexing, MTIA, MTI-A, MTIFL, MTI-FL, MTIX, MTI-X, semantic indexing, semi-automated indexing, and semi-automatic indexing. We also conducted supplementary online browsing to identify additional grey literature.

Summary Table of our supplemental / GL searching

Type of searching / sources:	Number of Documents
1. Searching / online browsing of seven (7) key journals	29
2. NLM Websites	53
3. Health sciences library associations and conference websites	14
4. Open repositories and websites	38
**Total number retrieved:**	134

1. **Searching / online browsing of seven (7) key journals (29 documents)**

Journal	Search yield
Journal of the European Association for Health Information and Libraries (from 2019–2024)<br>https://ojs.eahil.eu/J EAHIL/issue/archive	**n = 4**
Evidence Based Library and Information Practice (from 2013–2024)<br>https://journals.library.ualberta.ca/eblip/index.php/EBLIP/issue/archive	**n = 2**
Journal of the Canadian Health Libraries Association (from 2004–2024)<br>https://journals.library.ualberta.ca/jchla/index.php/jchla/issue/archive	**n = 8**
Journal of the Medical Library Association (from 2000–2024)<br>https://jmla.mlanet.org/ojs/jmla/issue/archive	**n = 6**
Journal of eScience Librarianship (from 2012–2024)<br>https://publishing.escholarship.umassmed.edu/jeslib/issues/	**n = 1**
Medical Reference Services Quarterly (from 2000–2024)<br>https://www.tandfonline.com/loi/wmrs20	**n = 7**
Hypothesis (from 2009–2024)<br>https://journals.indianapolis.iu.edu/index.php/hypothesis/issue/archive	**n = 1**
	**29 papers**

2. **NLM Websites (53 documents)**

- 1) We searched **NLM Technical Bulletins** from 2000-2024: <https://www.nlm.nih.gov/pubs/techbull/back_issues.html>
  - **Search yield n=12**
- 2) We searched transcripts/résumés of **NLM PubMed Office Hours** from 2022-2024: <https://learn.nlm.nih.gov/documentation/training-packets/T000221112/?_gl=1*mdmpa8*_ga*MTQ0OTc2NTczLjE3NjY5MzIwMTk.*_ga_7147EPK006*czE3NzEyNTg3NjAkbzE0JGcxJHQxNzcxMjU5MDkxJGo1O

CRsMCRoMA..*_ga_P1FPTH9PL4*czE3NzEyNTg3NjAkbzE0JGcxJHQxNzcxMjU5MDkxJGo1OSRsMCRoMA..>
  - **Search yield n=11 documents.**
- 3) We searched the website of **NLM's Lister Hill National Center for Biomedical Communications** https://lhncbc.nlm.nih.gov and its archival equivalent on the WayBack Machine: https://wayback.archive-it.org/7867/20241213191923/https://lhncbc.nlm.nih.gov/ii/tools/MTI.html
  - **Search yield n=30 documents.**

3. **Health sciences library associations and conference websites (14 documents)**
- We searched for meeting posters and conference abstracts at the following websites:
  - Canadian Health Libraries Association (CHLA/ABSC) from 2001-2024 <https://www.chla-absc.ca/CHLA_FA_LIST_3815985.php>,
    - **Search yield n=4 documents**
  - European Association for Health Information and Libraries (EAHIL) from 2001-2024 <https://eahil.eu/events/>
    - **Search yield n=3 documents**
  - Medical Library Association (MLA) Proceedings from 2000-2024: https://www.mlanet.org/meetings-events/past-and-future-mla-conferences/
    - **Search yield n=7 documents**
  - **Total = 14**

4. **Open repositories and websites (38 documents)**
- We also searched the following websites:
  - **Figshare** <https://figshare.com/> **Search yield n=4 documents**
  - **Zenodo** <https://zenodo.org/> **Search yield n=3 documents**
  - **Open Science Framework (OSF)** <https://osf.io/> **Search yield n=11 documents**
- We also searched the following e-print archives:
  - arXiv.org e-Print archive https://arxiv.org **search yield = 11**
  - medRxiv.org - preprint server https://www.medrxiv.org **search yield = 9**
