# Supplementary material for: Automated indexing in MEDLINE and the Medical Text Indexer (MTI), 2000–2025: a scoping review
Source: J Med Libr Assoc. 2026 Jul 14;114(3):191–207. doi: 10.5195/jmla.2026.2406 (PMC13367316; doi:10.5195/jmla.2026.2406)
Supplement: Supplementary file 3 — Appendix C: Definition(s) of Study Methods [file jmla-114-3-191-s03.pdf]

Appendix C: Definition(s) of study methods

- Comparative Study – comparison of outcomes, results, responses, etc. for different techniques, approaches or inputs (MeSH pt) https://www.nlm.nih.gov/mesh/pubtypes.html
- Evaluation Study – publications determining the effectiveness or utility of processes, personnel, and equipment (MeSH pt) https://www.nlm.nih.gov/mesh/pubtypes.html
- Mixed Methods Research – "...mixed methods research is the type of research in which a researcher or team of researchers combines elements of qualitative and quantitative research approaches (e. g., use of qualitative and quantitative viewpoints, data collection, analysis, inference techniques) for the broad purposes of breadth and depth of understanding" (23) https://pmc.ncbi.nlm.nih.gov/articles/PMC5602001
- Qualitative Description Studies – qualitative description publications have as their goal a comprehensive summary of events in the everyday terms of those events. Researchers conducting qualitative descriptive work stay close to their data and to the surface of words and events. Qualitative descriptive designs typically are an eclectic but reasonable combination of sampling, and data collection, analysis, and re-presentation techniques. Qualitative descriptive study is the method of choice when straight descriptions of phenomena are desired. (22) https://pubmed.ncbi.nlm.nih.gov/1
