# Supplementary material for: Automated indexing in MEDLINE and the Medical Text Indexer (MTI), 2000–2025: a scoping review
Source: J Med Libr Assoc. 2026 Jul 14;114(3):191–207. doi: 10.5195/jmla.2026.2406 (PMC13367316; doi:10.5195/jmla.2026.2406)
Supplement: Supplementary file 4 — Appendix D: Most Cited NLM and Other Researchers (2000–2025) [file jmla-114-3-191-s04.pdf]

Appendix D. Most cited NLM and other researchers (2000-2025)

Researcher	# of publications	Percentage of total
Mork JG	29	45%
Aronson AR	26	41%
Demner-Fushman D	14	22%
Neveol A	11	27%
Jimeno-Yepes AJ	8	13%
Rae AR	5	8%
Humphrey SM	5	8%
Shooshan SE	5	8%
Fernandez-Llimos F	4	6%
Rogers WJ	4	6%

Note: Automated indexing research from 2000-2025 was led by a core group of indexing specialists and experts affiliated with or working at the NLM, with nine of the top ten researchers co-authoring two-thirds (66%) of all publications (n=42). Collaboration networks are dense with Mork, Aronson, Demner-Fushman, Neveol, and Jimeno-Yepes frequently collaborating and co-authoring. Among the top ten, only pharmacy researcher Fernandez-Llimos (n=4, 6%) had no NLM affiliation.
