# Supplementary material for: Automated indexing in MEDLINE and the Medical Text Indexer (MTI), 2000–2025: a scoping review
Source: J Med Libr Assoc. 2026 Jul 14;114(3):191–207. doi: 10.5195/jmla.2026.2406 (PMC13367316; doi:10.5195/jmla.2026.2406)
Supplement: Supplementary file 5 — Appendix E: Publication Venue [file jmla-114-3-191-s05.pdf]

Appendix E. Publication venue

Journal name	Count
Journal of the Medical Library Association	4
BMC Bioinformatics	3
Journal of Biomedical Informatics	3
Research in Social & Administrative Pharmacy	3
JAMIA Open	2
Bioinformatics	2
Advances in Classification Research Online	1
Frontiers in Research Metrics and Analytics	1
Health Information and Libraries Journal	1
International Journal of Medical Informatics	1
International Journal of Pharmacy Practice	1
Journal of Biomedical Semantics	1
Journal of Computer Science & Engineering	1
Journal of the Association for Information Science & Technology (JASIST)	1
PLoS One	1
Studies in Health Technology and Informatics	1
**Total**	27
