# Supplementary material for: Automated indexing in MEDLINE and the Medical Text Indexer (MTI), 2000–2025: a scoping review
Source: J Med Libr Assoc. 2026 Jul 14;114(3):191–207. doi: 10.5195/jmla.2026.2406 (PMC13367316; doi:10.5195/jmla.2026.2406)
Supplement: Supplementary file 6 — Appendix F: Study Methods Distribution [file jmla-114-3-191-s06.pdf]

Appendix F. Study Methods Distribution

**Study Method**	**Count**	**Percentage of total**
Evaluation Study	34	54%
Qualitative Description	16	25%
Comparative Study	7	11%
Mixed Methods	7	11%
**Total**	**64**	

Note. Each publication was classified by its primary methodological approach. Published reports and commentaries were grouped under qualitative description.
