# Supplementary material for: Automated indexing in MEDLINE and the Medical Text Indexer (MTI), 2000–2025: a scoping review
Source: J Med Libr Assoc. 2026 Jul 14;114(3):191–207. doi: 10.5195/jmla.2026.2406 (PMC13367316; doi:10.5195/jmla.2026.2406)
Supplement: Supplementary file 7 — Appendix G: BERT-Based Models [file jmla-114-3-191-s07.pdf]

Appendix G. BERT-based models

Model	Description
BERT Model (27,31,42)	BERT is a pre-trained language model developed by Google in 2018 (87) and used to improve indexing, classification, and textual understanding. BERT embeddings enable semantic similarity, word-sense disambiguation, and learning-to-rank approaches, supporting MeSH assignment beyond simple lexical matching.
BioBERT & PubMedBERT (31,39,41,42,44)	BioBERT and PubMedBERT adapt BERT for the biomedical domain by pretraining on PubMed abstracts and full-text articles in PubMed Central. They generate embeddings that capture domain-specific terminology, acronyms, and conceptual relationships. (Both models are resource-intensive to train and deploy, and consume significant computational power and energy.)
DistilBERT (27)	DistilBERT is a distilled version of BERT that maintains much of BERT's semantic capabilities while reducing model size and computational costs. DistilBERT and other compressed variants retain BERT's semantic capabilities while reducing model size, inference time, and power requirements, offering a more energy-efficient alternative for large-scale indexing tasks.
SciBERT (44)	SciBERT is pretrained on a large corpus of scientific literature covering biomedical and physical sciences. It enhances semantic similarity and document–concept matching, improving automated indexing and information retrieval. (Pretraining SciBERT requires high-performance computing resources, resulting in considerable energy consumption and environmental impact.)

Note: Training and deploying BERT models is computationally intensive, and requires substantial power and memory resources, which raises environmental and operational considerations (90).
