# Supplementary material for: Automated indexing in MEDLINE and the Medical Text Indexer (MTI), 2000–2025: a scoping review
Source: J Med Libr Assoc. 2026 Jul 14;114(3):191–207. doi: 10.5195/jmla.2026.2406 (PMC13367316; doi:10.5195/jmla.2026.2406)
Supplement: Supplementary file 8 — Appendix H: Table: Summaries of 64 Publications [file jmla-114-3-191-s08.pdf]

Appendix H

Table 1. Summaries of 64 publications (most recent first)

Reference	Objective	Publication Type / Country	Study Methods	Algorithm	Key Findings
(24) Allen et al, 2025.	To disseminate an author checklist to improve accurate indexing	Poster / United States	Mixed methods	MTIX[1]	Misleading, negative and imprecise language, and "cute" titles may confuse MTIX algorithm. Authors should work to improve discoverability by writing clear, literal titles and abstracts.
(25) Amar-Zifkin et al, 2025.	To evaluate appropriateness of algorithmic indexing of MEDLINE records	Journal Article / Canada	Evaluation study	MTIA	53% of records were indexed adequately; 47% had issues. Future research should examine indexing for populations (and other characteristics from full-text) and its downstream effects on information retrieval.
(26) Askin et al, 2025.	To evaluate search filter 'exp animals/not humans.sh' performance due to automated indexing	Journal article / Canada	Evaluation study	MTIA	Assignment of human check tag by MTIA alone had higher error rate compared to curated records. Pending improvements, searchers should test impacts of filters in searching.
(27) Cid et al, 2025.	To examine whether assignment of Publication Types (PTs) can be improved using transformer models	Preprint / United States	Evaluation study	MTIX	The "Rules-based MTI" perpetuated issues for publication types. Results demonstrate the potential of transformer-based models to significantly improve PT tagging accuracy.

[1] For definitions, see Appendix A - Glossary of terms and abbreviations.

(28) Epp et al, 2025	To evaluate MTI false exclusion of human studies in search filters compared to manual indexing	Journal article / Canada	Evaluation study	MTIA, MTIX	MTIX was less likely to mis-index human studies (April 2024-present) than MTIA (2019-Mar 2024). Exercise caution with use of human studies filter in records from 2019-2024.
(29) Fernandez Llimos et al, 2025	To evaluate consistency of MeSH terms in the same articles published more than once in pharmacy journals	Journal article / Portugal	Evaluation study	MTIA	Pharmacists expressed concerns about indexing and its impact on searching. Notably, one pharmacy guideline published in several journals received different indexing each time.
(30) Gram et al, 2025.	To examine how MeSH “Overdiagnosis” was applied to records from 2020 to 2023	Journal article / Denmark	Qualitative Description	MTIA	"Overdiagnosis" [MeSH] was often misapplied but using “overdiagnosis” in title or abstract will help improve accurate indexing.
(31) Islamaj et al, 2025.	To evaluate integrating AI-based gene–article linking affects gene linking	Journal article / United States	Mixed methods	MTIX	Key changes helped curators maximize GNorm2 and improved the algorithm to cover 135 species of genes including viral and bacterial genes, based on a survey.
(32) Philippopoulos 2025.	To examine systemic exclusion and gaps in MEDLINE indexing regarding intersex-related research	Conference paper / Canada	Evaluation study	MTIX	The MeSH term “Intersex Persons” (2020) was assigned inconsistently or omitted in both automated and human indexing.
(33) Wilson 2025.	To identify errors in records with MeSH term Malus, the genus name for apple trees	Journal article / United States	Qualitative Description	MTIX	7.9% of 1,705 records were incorrectly indexed with MeSH term Malus. Most common error was due to “apple” used in similes, metaphors, and idioms (80, or 59.2%), with the next most common error being when apple was present in a name or term (50, or 37%).
(34) Fernandez Llimos et al, 2024	To compare indexing methods (manual, automated, curated) on MeSH assignment in pharmacy journals	Journal article / Portugal	Comparative study	MTIA	Pharmacy practice journals had significantly fewer MeSH terms than general medical journals; automated indexing assigned fewest.

(35) Moore et al, 2024.	To compare MTI's disease MeSH against manual indexing in MEDLINE for seven document types	Journal article / United States	Comparative study	MTIA	MTI's recall and precision showed it reliably identified diseases, with few errors, in grant descriptions, patent texts, and drug indications. MTI is a potentially useful tool for researchers wishing to categorize texts from a variety of sources into disease categories.
(10) NLM. MTIX, 2024.	To announce NLM's plans to move to automated indexing using the MTIX in 2024	Report / United States	Qualitative Description	MTIX	MTIX-indexed articles captured concepts, and had higher F-scores for publication types, and check tags. MTIX outperforms MTIA by "understanding" more complex representations of concepts.
(36) Tonin et al, 2024.	To evaluate MeSH accuracy in patient simulation articles	Journal article / Brazil	Evaluation study	MTI	Inconsistent use of terms related to patient simulation was identified in scientific articles, alongside inaccuracies in the assignment of MeSH terms to articles in the 'simulated patient' field by NLM cataloguers.
(37) Chen et al, 2023.	To compare MeSH assignment in MTI with a sample of previously indexed records by human indexers	Journal article / Canada	Evaluation study	MTI	MTI missed or misapplied age and sex check tags. Relevant terms with lower rankings were often non-medical or allied health in nature.
(38) Guo et al, 2023	To understand how automatic indexing affects search performance	Preprint / United States	Evaluation study	MTIA	It is unclear how human review/curation efforts are combined with automatic term assignments in the 'MTI-Auto' system. Complementing automatic indexing with manual indexing can prevent low-performing terms from impacting query-level performance.
(39) Krithara et al, 2023.	To describe evolution of MTI and role of BioASQ (2013)	Journal article / United States	Evaluation study	MTI, MTIFL, MTIR, MTIC	NLM has a team of experienced curators to review and re-index specific types of articles and random samples. Any corrections made by the curators are fed back into improving MTIA and corrected on any affected articles.

(40) Rae et al 2023.	To test indexer assignment dataset for articles indexed between 2011 and 2019	Journal article / United States	Evaluation study	MTIA	The NLM indexer assignment dataset is a large dataset containing over 4.2 million article assignments to 144 different indexers. The dataset was shown to be a useful test bed for expert matching and assignment algorithms.
(9) NLM. MEDLINE 2022.	To announce NLM's plans to move to automated indexing using the MTIA in 2022	Report / United States	Qualitative Description	MTIA	MTIA transition includes an integration of deep learning models but human indexers at NLM will continue to perform quality assurance.
(41) Rae et al, 2021.	To develop neural network for attachment of subheadings to main headings	Conference paper / United States	Evaluation study	MTIA	The best performing method outperforms the current MTI with 3.7% improvement in precision and 27.6% improvement in recall. Authors conduct a manual review of false positive predictions and 70% were found acceptable.
(42) Rae et al, 2021.	To investigate neural text ranking approach for automatic MeSH indexing	Conference paper / United States	Evaluation study	MTIA, MTIR	The neural text ranking approach was shown to have good performance, and multi-stage text ranking was found to boost the F-score by about 5% points.
(43) Rae et al, 2021.	To develop a machine learning classifier to semi-automate identification of biomedical articles	Conference paper / United States	Evaluation study	MTI	The system was shown to offer significant time and cost savings by allowing indexers to discard 54% of articles that were unlikely to require indexing.
(44) Savery et al, 2020.	To evaluate eleven chemical entity recognition systems, and to identify a tool that effectively recognizes chemical entities for indexing	Conference paper / United States	Evaluation study	MTI	SciBERT ensemble may provide the greatest contribution to MTI; in general, the BERT architecture pre-trained on biomedical data and fine-tuned on chemical entity mentions outperforms other approaches.

(45) Rae et al, 2019.	To evaluate deep learning approach (convolutional neural network) vs the MTI	Conference paper / United States	Evaluation study	MTI	The CNN model demonstrated competitive performance and outperforms NLM's Medical Text Indexer (MTI) by about 3%.
(46) Mork et al, 2017.	To discuss how well MTI performs and to evaluate its utility	Journal article / United States	Qualitative Description	MTI	The role of MTI at NLM will expand into new areas, further reinforcing the idea that MTI is increasingly useful and relevant.
(47) Demner-Fushman et al, 2016	To report on automated indexing at NLM, its significance and ongoing development	Report / United States	Qualitative Description	MTI, MTIFL, MTIA	The MTI was initiated to address practical NLM needs, and developed into a fully-fledged research project. BioASQ has driven improvements but human indexing is still considered the "gold standard".
(48) Zavorin et al, 2016.	To present a Learning to Rank (LTR) method at BioASQ	Conference paper / United States	Evaluation study	MTI	Integration of LTR as a boosting component of the MTI improved its overall performance and showed significant gains in both precision and recall for specific classes of MeSH headings.
(49) Demner-Fushman et al, 2015	To explore potential of full-text articles in improving indexing precision for study subjects	Conference paper/ United States	Evaluation study	MTIFL	Full-text improved MTI. The results indicate the need for further development of methods capable of leveraging the full text for indexing.
(50) Minguet et al, 2015.	To examine pharmacy-specific MeSH assignments in pharmacy journals	Journal article / Spain	Qualitative Description	MTI	Pharmacy has fewer MeSH compared with nursing and dentistry. Collaboration is needed between pharmacy professionals and indexers to improve indexing accuracy and depth.
(51) Mork et al, 2014.	To provide an update on MTI functionality and performance since first BioASQ Challenge	Conference paper / United States	Qualitative Description	MTI	BioASQ drives MTI enhancements. Filtering for ambiguity, weighting, ranking, and journal context improved indexing accuracy.

(52) Mork et al, 2013.	To provide an overview of functionality, performance and evolution of MTI	Conference paper / United States	Qualitative Description	MTI	MTI provided recommendations for over 93% of total number of citations indexed in 2012. Authors use human indexing as a gold standard and compare it against the MTI.
(53) Jimeno-Yepes et al, 2013	To use titles and abstracts (or full-text) to create summaries (50–150 words)	Journal article / United States	Evaluation study	MTI	Article summaries improved MTI indexing. Graph-based summaries increased precision but reduced recall; frequency-based summaries did the opposite.
(54) Yepes et al, 2013.	To evaluate machine learning algorithms' ability to recommend publication types	Conference paper / United States	Evaluation study	MTI	MTI performed poorly for publication types; machine learning improved assignments. Logistic regression outperformed Naive Bayes.
(55) Yepes et al, 2013.	To extend MTI adding a diverse set of MeSH using examples where MTI had poor performance	Conference paper / United States	Comparative study	MTI	Authors compared MTI with Naive Bayes, SVM, AdaBoost, and voting combinations; algorithms and machine learning improved performance.
(56) Jimeno-Yepes et al, 2012	To select the best indexing method per document since no single approach works best for all articles.	Journal article / United States	Evaluation study	MTI	The described framework allowed comparison of alternative indexing strategies, and an automated way of deciding on an optimal strategy for use with the MTI.
(57) Jimeno-Yepes et al, 2012.	To examine how machine learning can be used to improve the MTI	Conference paper/ United States	Qualitative Description	MTI	ML can improve annotations recommended by MTI and achieves better performance despite the fact that each MeSH assignment tasks present different challenges (or "behavior").
(58) Herskovic et al, 2011.	To examine automatic indexing using MEDRank to identify major headings	Journal article / United States	Evaluation study	MTIA	Adding MEDRank to the MTI significantly improved retrieval of core concepts in MEDLINE abstracts and more closely matched human expectations compared to MTI without MEDRank.

(59) Jimeno-Yepes et al, 2011.	To describe an approach using MeSH triage rules and a false-positive filter using statistical learning algorithms.	Conference paper / United States	Evaluation study	MTI	Bottom-up approach scanned abstracts for keywords, mapped to MeSH, then trained classifiers on MEDLINE data, improved sensitivity of MeSH recommendations.
(60) Jimeno-Yepes et al, 2011	To create word sense disambiguation (WSD) dataset using Unified Medical Language System (UMLS) & manually-indexed records	Journal article / United States	Evaluation study	MTI	The dataset allows evaluation of WSD algorithms; compared to previously existing data sets, WSD contains a larger number of biomedical terms/abbreviations and covers the largest set of UMLS Semantic Types.
(61) Demner-Fushman et al, 2010.	To test UMLS content views to filter the Metathesaurus; to improve MTI's precision and recall by reducing irrelevant or overly ambiguous mappings	Journal article / United States	Evaluation study	MTI	MTI improved indexer consistency and reduced workload. Performance was boosted by adding clustering, machine-learning and rules for journals and check tags. Indexing noise was reduced by filtering through the UMLS.
(62) Neveol et al, 2010.	To examine use of author-supplied keywords and their value in MeSH assignments	Conference paper / United States	Evaluation study	MTI	Author keywords enhanced MTI assignments, and revealed new MeSH candidates. Over 60% of author keywords can be linked to a closely related indexing term.
(63) Humphrey et al, 2009	To compare how rules-based (Journal Descriptor Indexing) and machine learning-based systems assign MeSH	Journal article / United States	Comparative study	MTI	Rule-based (deterministic) and statistical (machine-driven, probabilistic)- based indexing were compared against human indexing. Hybrid approaches improved workflow and performance.
(64) Neveol et al, 2009.	To review Trieschnigg et al. (2009) on machine learning-based MeSH classification	Commentary / United States	Qualitative Description	MTI	Trieschnigg et al tests were difficult to reproduce and results did not confirm previous work in automated indexing.

(65) Neveol et al, 2009.	To report on six different methods in resolving subheading attachment issues	Journal article / United States	Mixed methods	MTI	Six MTI components were evaluated individually and combined. Combined methods improved F-score, though errors remain.
(66) Trieschnigg et al, 2009.	To compare six systems in MeSH assignments	Journal article / United Kingdom	Comparative study	MTI	kNN classifier outperformed MTI in ranking MeSH terms from titles and abstracts. Neveol et al. (2009) noted methodological limitations.
(67) Neveol et al, 2008.	To evaluate inductive logic programming to infer indexing rules to produce automated indexing recommendations	Journal article / United States	Mixed methods	MTI	Inductive logic programming increased MTI precision, achieving indexer-approved accuracy, higher recall and minimal precision loss with subheadings.
(68) Aronson et al, 2008.	To evaluate MTI progress and report on possible ideas for improvement	Report / United States	Evaluation study	MTI	Indexers made 1000+ requests of MTI daily. Recent work has focused on expanding MTI's capabilities and its accuracy and usefulness to NLM indexers.
(69) Ruiz et al, 2008.	To provide an overview of the state-of-the-art in automatic and computer aided indexing systems	Report / United States	Mixed methods	MTI	Final recommendations and "best practices" will be presented and discussed among panel members and the audience..
(70) Neveol et al, 2007.	To evaluate three methods for assigning MeSH main-heading and subheading pairs	Conference paper / United States	Evaluation study	MTI	The best overall performance is obtained for the subheading genetics (70% precision and 17% recall with post-processing rules, 48% precision and 37% recall with the dictionary-based method).

(71) Neveol et al, 2007.	To evaluate two statistical methods of producing MeSH indexing recommendations for the genetics literature	Conference paper / United States	Evaluation study	MTI	Statistical methods can be used to produce recommendations involving subheadings. Domain specific representation of documents can contribute to enhancing recall.
(72) Neveol et al, 2007.	To address automatic attachment of subheadings to MeSH main headings	Conference paper / United States	Mixed methods	MTI	For main heading/subheading pair recommendations, the best precision is obtained with a post-processing rule method (58%) while the best recall is obtained by pooling all methods (64%).
(73) Ruiz et al, 2007	To evaluate the MTI using a survey and conducting interviews with indexers	Report / United States	Mixed methods	MTI	MTI yielded time savings. Less experienced indexers used it more; 40% reported productivity gains; 75% lacked confidence in recommendations.
(74) Humphrey et al, 2006.	To compare journal descriptor indexing (JDI) to categorize text and journal descriptors or UMLS semantic types	Conference paper/ United States	Comparative study	MTI	The JDI tool uses a methodology based on statistical word associations from a training set of MEDLINE citations to which are imported the journal descriptors (JDs) corresponding to journal unique identifiers in the citations.
(75) Neveol et al, 2006.	To evaluate MTI recommendations for biomedical journal articles on a random set of MEDLINE citations	Conference paper / United States	Evaluation study	MTI	MTI-assigned MeSH terms were shown to reach a high semantic similarity with human indexing and capture inter-concept relationships.
(76) Gay et al, 2005	To evaluate inclusion of full-text in automated indexing	Conference paper/ United States	Evaluation study	MTI	The resulting model provides indexing significantly better (7.4%) than what is currently achieved using only titles and abstracts.

(77) Kim et al, 2005.	To estimate probability a given citation should receive a new heading	Conference paper / United States	Evaluation study	MTI	New topics are “MeSH-invisible”; emerging topics (genomics, nanotechnology) are under indexed. Bayesian methods help human indexers keep pace with new terms.
(78) Neveol et al, 2005.	To evaluate indexing for English-language (MetaMap) and French-language (VUMeF) texts using MAIF (MeSH Automatic Indexer for French)	Conference paper / United States	Evaluation study	MTI	MTI outperforms MAIF in recall and F-scores but faces multilingual indexing challenges; MAIF optimizes performance by recommending adaptive descriptor counts for each resource.
(79) Aronson et al, 2004.	To evaluate performance of MTI and Data Creation and Maintenance System (DCMS)	Journal article / United States	Mixed methods	MTI	MTI performance varied due to subject matter and journal. Indexers reported DCMS was fully or partially helpful.
(80) Gay et al. National Library of Medicine, 2002.	To report about indexers' evaluation of MTI	Report / United States	Qualitative Description	MTI	Reports on the input of indexers on whether indexing terms suggested by the MTI facilitates the work of indexers.
(81) Aronson, 2001.	To describe MetaMap in semi and fully automatic indexing	Conference paper/ United States	Qualitative Description	MTI	MetaMap mapped to UMLS but struggled with chemical names, abbreviations, and numbers. Word sense disambiguation and semantic classification may improve performance.
(82) Kim et al, 2001.	To evaluate how indexing quality may be evaluated using machine learning	Conference paper / United States	Evaluation study	MTI	The results showed that machine-assigned MeSH are on par with humanly-assigned MeSH terms on three test sets.

(83) Aronson et al, 2000.	To describe NLM's Indexing Initiative (IND) to automate indexing	Conference paper/ United States	Qualitative Description	MTI	NLM sought to improve MeSH recommendations. Automated (or partly automated) indexing will succeed when it matches human indexing.
(84) Bodenreider, 2000.	To describe a method in which the semantic relationships between UMLS concepts are exploited for the purpose of classification	Conference paper/ United States	Qualitative Description	MTI	When applied to the automatic classification of condition terms into broad disease categories, this method assigned relevant categories to 92% of the 1823 condition terms encountered. 135 (7%) failed to be classified and 14 (.77%) were misclassified.
(85) Humphrey et al, 2000.	To describe journal descriptor (JD) indexing at the journal level using 127 descriptors, and applying statistical methods that associate journal indexing with text words in a training set of MEDLINE citations.	Journal article / United States	Comparative study	MTI	Semantic type indexing may convey a unique slant of a document's content not normally represented in standard indexing vocabularies. Use of ST indexing to rank retrieved output is mentioned as a possible application.
